# Technical Details of Lateral Tenodesis at the Fascia Lata: A Systematic Review of the Literature

**DOI:** 10.3390/jcm14217613

**Published:** 2025-10-27

**Authors:** François Laudet, Thibaut Noailles, Christian Lutz, Alexandre Hardy

**Affiliations:** 1 Département de Chirurgie Orthopédique, Hôpital Robert Boulin, 112 rue de la Marne, 33500 Libourne, France; 2 Département de Chirurgie Orthopédique, Polyclinique de Bordeaux Nord, 15/35 rue Claude Boucher, 33000 Bordeaux, France; noaillesthibaut@yahoo.fr; 3 Institut de Chirurgie Orthopédique et Sportive à Strasbourg, 35 Avenue du Rhin, 67100 Strasbourg, France; 4 Clinique du Sport Paris V, 36 Boulevard Saint Marcel, 75005 Paris, France

**Keywords:** ACL reconstruction, lateral tenodesis, Lemaire plasty, fascia lata

## Abstract

**Background/Objectives**: Anterior cruciate ligament (ACL) reconstruction stabilizes the knee and treats associated lesions. Control of rotational laxity can be optimized by an extra-articular plasty, historically known as the Lemaire plasty or tenodesis at the fascia lata. The risk of iterative rupture is reduced, by stabilising anterior translation and tibial internal rotation. In the literature, many different technical details are described, for example regarding the position and the method of femoral fixation. Although these parameters are fundamental to achieving graft isometry and avoiding overconstraint, no consensus or standardized recommendations have yet been established. The aim of the study was to summarize the position of fixation of a lateral tenodesis to the fascia lata, the degrees of flexion and rotation during fixation, its mode of fixation and its passage in relation to the lateral collateral ligament (LCL). **Methods**: In April 2024, a systematic review was conducted by two independent authors to identify studies describing lateral tenodesis with fascia lata with details about femoral fixation position, method of fixation, the graft’s passage relative to the lateral collateral ligament and flexion/rotation during fixation. From 111 records initially identified, 22 studies met the inclusion criteria. **Results**: Most authors described passing the graft beneath the LCL to achieve controlled anisometry. The preferred femoral fixation point was proximal and posterior to the lateral epicondyle, with fixation performed at approximately 30° of flexion and neutral tibial rotation. Various fixation methods have been reported, including staples, screws, cortical buttons, and anchors, but no biomechanical evidence demonstrated the superiority of one technique over another. **Conclusions**: Lateral tenodesis at the fascia lata is a well-established adjunct to ACL reconstruction, providing additional control of rotational laxity. While consistent trends are emerging regarding graft passage and femoral fixation landmarks, technical heterogeneity persists, and no definitive guidelines currently exist. Standardization of these parameters through high-level clinical and biomechanical studies is warranted to optimize outcomes and reduce variability in surgical practice.

## 1. Introduction

Anterior cruciate ligament (ACL) reconstruction with autograft is one of the most common procedures in orthopedic sports traumatology [1,2,3], particularly among young and athletic populations. The primary goal is to restore knee stability and enable patients to return to sports (including pivoting sports) while minimising the risk of graft rupture. Recent studies have described structural and molecular differences between the epiligament of the anterior cruciate ligament and extra-articular ligaments such as the medial collateral ligament, which may explain why the anterior cruciate ligament heals less spontaneously than the medial collateral ligament [4]. These observations, known as ‘the epiligament theory’, shed light on the contrasting healing trajectories between knee ligaments. However, despite technical improvements, a proportion of patients continue to describe residual anterolateral instability. This instability not only predisposes to graft failure but also compromises functional outcomes and return to sports. To address this, combined procedures with reinforcement of the anterolateral structures, such as reconstruction of the anterolateral ligament (ALL), have been proposed. These adjunctive techniques provide improved rotational control [5,6,7,8] and can reduce the risk of iterative rupture by a factor of 5 [9,10].

Historically in 1967, the first attempt to control rotational laxity was made by Lemaire who described an anterolateral plasty, also known as lateral extra-articular tenodesis or Lemaire plasty, using a strip of fascia lata [11]. The fascia lata is well-documented anatomical structure [12] with a recognized biomechanical role in rotational control. It shifts part of the stresses transmitted to the anterior cruciate ligament extra-articularly, thus reducing rotational laxity [13]. Its use as an autograft has been described in combined reconstructions (anterior cruciate ligament and anterolateral ligament) through a single femoral tunnel [14,15].

Whatever the anterior cruciate ligament reconstruction technique, various lateral extra-articular tenodesis variants have been described. For all of them, the purpose was to limit anterior tibial translation and internal rotation. Lateral extra-articular tenodesis typically uses a proximally detached strip of fascia lata, maintaining its distal insertion behind Gerdy’s tubercle, thus reinforcing the knee extra-articularly. This dual intra- and extra-articular concept helps restore the knee’s kinematics [12,16,17] while protecting the intra-articular graft. For optimal results, femoral fixation should ideally occur in an anatomical position to ensure favourable isometry and effective rotational control near extension [18].

In recent years, interest in anterolateral stability has markedly increased, with renewed attention to the anterolateral complex, which encompasses the iliotibial band, its deep Kaplan fibers and the anterolateral ligament. Contemporary biomechanical studies highlight the iliotibial band and its deep fibers as critical secondary stabilisers of internal rotation in anterior cruciate ligament deficient knees [16]. Renewed knowledge of their importance has contributed to promoting the resurgence of the lateral extra-articular tenodesis and related procedures in modern clinical practice [10,19,20,21].

Clinically, lateral extra-articular tenodesis such as the Lemaire tenodesis have regained prominence as an adjunct to anterior cruciate ligament reconstruction. Indications include young athletes practicing pivoting sports, revision ACL reconstruction, patients with generalized ligamentous laxity and those with high-grade pivot shift [22,23,24,25]. Recent systematic reviews and meta-analyses confirm its efficacy: the addition of LET to ACL reconstruction significantly reduces graft rupture, improves pivot-shift control and enhances return-to-sport rates, although at the cost of slightly higher reoperation rates in some series [26,27,28]. These findings underline the importance of lateral extra-articular tenodesis as a protective and complementary procedure, especially in high-risk populations.

Despite growing evidence, technical aspects remain debated, including the ideal femoral fixation site (proximal and posterior to the lateral epicondyle versus other landmarks), the degree of flexion and tibial rotation at the time of fixation, the choice of fixation method (staples, screws, anchors, or cortical buttons) and whether the graft should pass superficial or deep to the lateral collateral ligament (LCL). The absence of standardized guidelines perpetuates variability in surgical practice and complicates comparisons across studies [20,21,22,23,24,25].

In this context, lateral tenodesis at the fascia lata represents a well-established but variably executed technique. The objective of this systematic review is to clarify its technical aspects and synthesize the available evidence, with a particular focus on four parameters: (1) femoral insertion; (2) degree of flexion and rotation during fixation; (3) fixation method; and (4) relationship of the graft to the LCL. We hypothesize that femoral fixation should be performed proximal and posterior to the lateral epicondyle at 30° of flexion and neutral tibial rotation, with the graft passing beneath the LCL and secured with a cortical anchor.

## 2. Materials and Methods

### 2.1. Search Strategy

The structure of this review followed the recommendations for systematic literature reviews [29,30]. The objectives, analysis methods and inclusion criteria followed the PRISMA (Preferred Reporting Items for Systematic reviews and Meta-Analysis) recommendations (more details in Supplementary Materials) and were determined prior to data collection. Between 1 April 2024 and 26 April 2024, a bibliographic search was conducted on Medline, Embase, Scopus and Cochrane using the MeSH keywords “tenodesis”, “extra-articular tenodesis” AND “iliotibial band”, “fascia lata” OR “Lemaire procedure”, “Lemaire tenodesis”, “modified Lemaire”.

An initial selection of studies was made based on the title and abstract by two independent authors followed by a full-text analysis of all selected articles to ensure no relevant articles were missed. The selected studies met the following criteria: (1) no publication date limit; (2) written in English or French; and (3) with an available abstract.

### 2.2. Inclusion Criteria

All articles reporting on the technical principles of lateral tenodesis at the fascia lata during combined anterior cruciate ligament and anterolateral ligament reconstruction were included.

The analysis focused on four criteria: femoral fixation location, degree of rotation and flexion during fixation, method of fixation and the relationship of the tenodesis to the lateral collateral ligament.

### 2.3. Exclusion Criteria

Articles describing isolated tenodesis, articles describing anterior cruciate ligament and anterolateral ligament reconstruction sing the same graft, articles lacking one of the four analysis criteria and articles not available in “full text” were excluded from the analysis.

### 2.4. Item Selection

**Figure 1:**
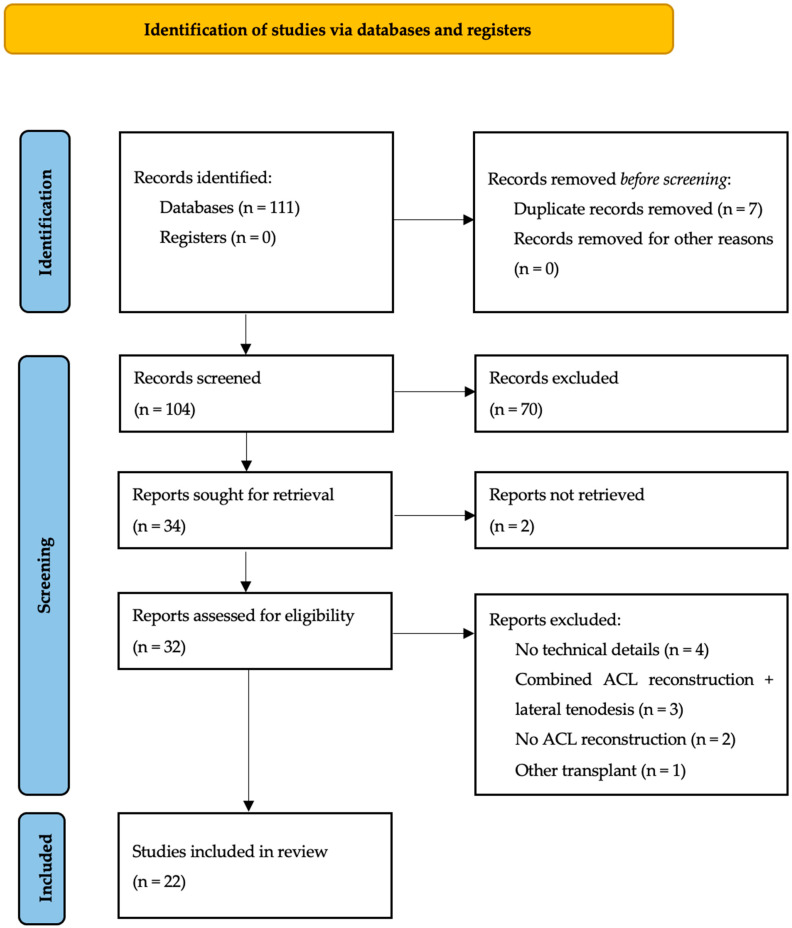
Flow diagram.

The search strategy (Figure 1) retrieved 104 articles, then selected 34 based on title and abstract, using keywords. Twelve were excluded. A total of 22 articles were included in the final analysis. The nomenclature used in the articles was: proximal/distal, anterior/posterior, superficial/deep.

## 3. Results

All results are summarized in Table 1.

### 3.1. Femoral Insertion

Half of the studies analyzed (11/22) proposed a proximal and posterior femoral insertion relative to the lateral epicondyle. Posteriorly, the reported distances to the lateral epicondyle are 3 mm [35,39], 5 mm [24,36] and 10 mm [42].

Proximally, they are 3 mm [35,39], 5 mm [24], 8 mm [36] and 10 mm [42]. Bernholt et al. [34] and Gali et al. [44] use distal Kaplan fibers as a reference, 31.4 mm from the lateral epicondyle proximally. Wasdev et al. [36] suggest four criteria for anatomical insertion: at the distal insertion of the Kaplan fibers, 15 mm proximal and 15 mm anterior to a grouping of vessels of the lateral epicondyle parallel to each other; at a fat pad 15 to 20 mm proximal and 15 mm posterior to the lateral epicondyle; at a bony prominence 20 mm proximal and 10 mm posterior to the lateral epicondyle; and 8 mm proximal and 4 mm posterior to the femoral insertion of the lateral collateral ligament. Schlichte et al. [38] performs femoral fixation in a pediatric population under the physeal after-image intensifier location. Puzzitiello et al. [21] and Anderson [1] describe a femoral insertion anterior to the epicondyle.

### 3.2. Fixing Position

Regarding tibial rotation during lateral tenodesis fixation, 17/22 authors propose neutral rotation. Stuyts et al. [33], Gomes et al. [25] and Leyes-Vence et al. [41] favoured a 15° lateral rotation.

For flexion, 13/22 authors fixed lateral tenodesis at 30° of flexion. Five authors out of 22 describe flexion around 60°. Two authors fix the tenodesis up to 90° flexion [32,33]. Moro et al. [42] perform full extension fixation in a single femoral tunnel. Koukoulias et al. suggest 10° fixation [43].

### 3.3. Femoral Fixation Methods

Different types of femoral fixation are proposed by the authors. For 10/22 authors, fixation is cancellous in a femoral tunnel with interference screws. For 6/22 authors, fixation is cortical using staples, all suture anchors or endobuttons, without creating a complete tunnel.

Lastly, in 6/22 cases, fixation was achieved by a simple soft-tissue suture, using either the traction sutures of the anterior cruciate ligament graft or the sutures of the endobutton used to fix the anterior cruciate ligament. In three studies [20,25,38], tenodesis suture is added to the chosen fixation system.

### 3.4. Tenodesis Passage in Relation to the Lateral Collateral Ligament

In 16/22 studies analyzed, the tenodesis passed under the lateral collateral ligament to ensure a ‘pulley’ effect. Only four studies [24,32,33,44] described a passage above the lateral collateral ligament. For two studies [31,40], the passage below the lateral collateral ligament is not specified.

It should provide a concise and precise description of the experimental results, their interpretation, as well as the experimental conclusions that can be drawn.

## 4. Discussion

This review focuses on the technical aspects of lateral extra-articular tenodesis using the fascia lata during anterior cruciate ligament reconstruction. Specifically, it addressed four technical details: femoral insertion, method of fixation, knee position during fixation and the relationship of the graft to the lateral collateral ligament. The synthesis of available data highlights both areas of emerging consensus and persistent heterogeneity in surgical practice.

Extra-articular reinforcement has been shown to reduce anterior tibial translation in the lateral compartment and improve control of anterolateral rotational instability following anterior cruciate ligament reconstruction [16]. Residual rotational instability remains a key predictor of unsatisfactory clinical outcomes and potential graft failure [45]. Reinforcement of the anterior cruciate ligament transplant by lateral extra-articular tenodesis reduces forces transmitted to the intra-articular reconstruction by approximately 40–45% [46], confirming its protective role. According to Getgood et al. [10], adding lateral tenodesis at the fascia lata reduces the iterative anterior cruciate ligament rupture risk by 25–40% at two years, as well as residual rotational laxity. The protective role of lateral reinforcement for the intra-articular graft is supported by studies from SANTY’s team on combined anterior cruciate ligament and anterolateral ligament reconstructions [15].

To achieve an anatomical insertion, several techniques have been reported to determine the optimal femoral insertion: percutaneous approaches, compass-based targeting, navigation and ultrasound localization. Wasdev et al. [36] described reproductible anatomical landmarks: Lemaire’s vessels, Kaplan’s fibers insertion on the lateral supra condylar ridge, fat pad and the lateral epicondyle. Most studies favour a position in relation to lateral epicondyle. The femoral insertion should be posterior and, most importantly, 3 to 10 mm proximal to the lateral epicondyle. This location ensures favourable isometry while minimizing overconstraint [23,36,39].

The literature is largely unanimous that fixation should be performed at approximately 30° of knee flexion in neutral tibial rotation [21,23,24,35,36,37,39,47]. This position promotes favourable anisometry, with the graft tensed in extension and relaxed in flexion. This allows control of rotational displacement without limiting physiological medial rotation. According to Engebretsen et al. [46], there is no significant difference in the force applied to the tenodesis between 30 and 90° of flexion. In cases with a single femoral tunnel used to both the anterior cruciate ligament graft and the tenodesis, some authors prefer fixation in nearly full extension to ensure optimal combined tension of both grafts [31,40]. To achieve favourable anisometry, fixation at 30° of flexion and neutral rotation is optimal.

A wide variety of fixation techniques are reported, including interference screws, cortical fixation (anchors or staples) and implant free suturing methods. Interference screws are most common. However, there is a risk of over-stressing the graft when it is buried, and a risk of tunnel convergence [48], which is increased in cases of revision. The risk of tunnel conflict is reduced when the lateral tenodesis tunnel is oriented anteriorly by at least 30° in the axial plane [49]. Goes et al. [34] describe a technique in which both the anterior cruciate ligament and tenodesis are fixed to the fascia lata using an interference screw in a single, dependent tunnel, thereby eliminating the risk of convergence. This is a demanding technique, requiring two grafts in opposite directions within a tunnel that must be precise both intra- and extra-articularly. Cortical fixation avoids additional tunnel and reduces the risk of convergence. Behrendt et al. [32] found no clinical or laxometric difference between screw and anchor fixation. Moreover, cortical fixation can reduce the length of the graft harvest. Stresses on the tenodesis are reduced with this type of fixation [35]. Some authors propose implant-free fixation, by suturing the graft to itself after creating a loop through the inter-muscular septum [22]. While this cost-effective technique is appealing, it is demanding, requiring five independent points of fixation and a rigidity that is difficult to determine. In the percutaneous technique described by Da silva Araujo et al. [24], fixation is achieved using the anterior cruciate ligament graft threads, without any proximal disinsertion. In this method, the anterior cruciate ligament out-in femoral tunnel must provide a proximal and posterior exit at the lateral epicondyle. In this technique, the graft is passed over the lateral collateral ligament. In pediatric cases, it is crucial to create a femoral tunnel below the physis to avoid the risk of epiphysiodesis. An image intensifier must be used to determine the optimal position for this tunnel [33].

The biomechanical objective of the anterolateral graft is to be relaxed in flexion, allowing unrestricted medial rotation, then tightened during extension to control rotational offset. Passage of the graft under the lateral collateral ligament promotes relaxation in flexion through a pulley effect, resulting in favourable anisometry [17]. As the native distal insertion at Gerdy’s tubercle is anterior, passing the graft under the lateral collateral ligament increases its length at the end of extension. According to Inderhaug et al. [16], a deep Lemaire procedure (under the lateral collateral ligament) at 20 N is superior to a superficial Lemaire procedure, which generates excess stress. Additionally, Neri et al. [13] reported over-stressing of the graft in medial rotation relative to the anterolateral ligament, regardless of whether it passed above or below the lateral collateral ligament. A modified Ellison procedure [50], where a strip of fascia lata is disinserted distally at Gerdy’s tubercle, passed under the lateral collateral ligament and reattached to the tibia, would allow physiological constraint.

Within the epiligament theory (a major source of cells and vessels during ligament repair), the medial collateral has a higher concentration of cells and vasculomyofibroblastic markers (VEGF, CD34, α-SMA) than the anterior cruciate ligament, which could explain its greater healing capacity [51]. In contrast, the epiligament of the anterior cruciate ligament shows different regional and quantitative characteristics that may limit revascularisation and cell recruitment after rupture, contributing to the failure of intrinsic healing [52]. Clinically, this supports approaches aimed at preserving or stimulating the epiligament (techniques that respect the tissue envelope) to improve anterior cruciate ligament healing [53].

Our findings align closely with emerging evidence in the literature, which underscores both the clinical value of lateral extra-articular tenodesis and the persistent technical variability that characterises its application. Recent meta-analyses have reinforced the clinical benefits of lateral extra-articular. Damayanthi et al. [27], in a meta-analysis of anterior cruciate ligament reconstruction with and without modified Lemaire lateral extra-articular, reported a significant reduction in graft failure (risk ratio 0.44) alongside improved Knee Injury and Osteoarthritis Outcome Score (KOOS) subscales across pain, activities of daily living, sports function and quality of life. Similarly, a systematic review by Zabrzyński et al. [54] showed that adding lateral extra-articular procedures to anterior cruciate ligament reconstruction significantly reduced rates of rotational instability and graft rupture, with functional outcomes equal or superior to isolated anterior cruciate ligament reconstruction. Biomechanical and functional data further support the lateral extra-articular’s protective role. Lemme et al. [55] demonstrated that lateral extra-articular effectively reduces anterior cruciate ligament graft forces and enhances knee stability, particularly in cases with increased posterior tibial slope. Kolin et al. [28] reported good patient outcomes and low graft failure rates during anterior cruciate ligament reconstruction with lateral extra-articular tenodesis, whether low or high knee flexion. These statements contrast with our study, showing a generalised fixation at 30°. Landrum et al. [56] confirmed that lateral extra-articular is a strong adjunct in young athletes, offering graft protection with minimal complication risk. Furthermore, Migliorini et al. [26] confirmed that adding a lateral tenodesis significantly reduces the risk of graft rupture and residual pivot shift, particularly in young athletes and high-risk populations. However, they also emphasized the considerable technical heterogeneity among included studies, with variations in fixation landmarks, graft tensioning angles and methods of femoral fixation, echoing the variability identified in our review. Importantly, while their work demonstrated the clinical benefits of lateral extra-articular tenodesis, it did not analyse in detail the technical parameters. In this regard, our study complements Migliorini’s findings by focusing precisely on the surgical nuances (femoral insertion, fixation method, knee position during fixation and graft passage relative to the lateral collateral ligament) thus bridging the gap between clinical outcomes and technical execution. Nevertheless, cadaveric studies by Herbst et al. [57] showed that in knees with an intact anterolateral capsule, the combination of anterior cruciate ligament reconstruction and lateral extra-articular may over-constrain internal rotation. These findings underline the importance of patient-specific indications and technical precision.

Lateral tenodesis at the fascia lata carries several potential complications, as summarized by Marshall et al. [58]. These include the risk of tunnel convergence, which can be minimized, or migration of the fixation system. The risk of postoperative hematoma ranges from 5 to 10%. To reduce this risk, the tourniquet is released at the end of the procedure to ensure hemostasis. Muscle herniation is rare, but has been described [59,60]. Joint stiffness or chronic pain may persist after surgery, especially if the lateral tenodesis is over-stressed. The risk of infection is low, ranging from 0 to 0.8% [7,61]. Finally, although rare, injury to the common fibular nerve may occur if the fascia lata is harvested too posteriorly. It is important to note that these surgeries can also have an impact on the surrounding soft tissues. In fact, the use of an arthroscopic pump can cause serum to spread into the soft tissues surrounding the joint. This is why some surgeons prefer to perform lateral tenodesis before performing arthroscopy.

This systematic review has several limitations. The majority of included studies had a low level of evidence, often simply descriptive technical notes restricting external validity. As a result, in these studies, there were no details about the populations benefiting from these surgeries (age, gender, physical activity, post-operative follow-up), clinical results or comparative analysis. No functional scores (IKDC, Lysholm) or post-operative complications were reported. No correlation between technical details and clinical outcomes could be established. Heterogeneity in surgical technique descriptions, including femoral fixation landmarks, angles of knee flexion during graft tensioning and relation to the lateral collateral ligament, precluded quantitative pooling. Thus, these techniques, which are specific to each surgeon, are difficult to extrapolate to a more general population.

Future research is required to conduct high-quality randomized controlled trials with standardized evaluation of rotational laxity, pivot shift and patient-reported outcomes. Comparative biomechanical studies are needed to determine the superiority of deep versus superficial graft passage relative to the lateral collateral ligament, in correlation with clinical outcomes. In addition, navigation-assisted or robotic surgery may optimize femoral tunnel positioning and standardise technical parameters. Integration of these findings into consensus statements could harmonise practice and improve outcomes for high-risk patients undergoing anterior cruciate ligament reconstruction with lateral extra-articular tenodesis.

## 5. Conclusions

Lateral tenodesis at the fascia lata offers rotational control and reduces the risk of iterative anterior cruciate ligament rupture. Although numerous fixation techniques have been described, this is a reliable procedure and it must meet precise technical criteria: the graft must pass under the lateral collateral ligament to achieve a pulley effect. The femoral insertion point must be proximal and posterior to the lateral epicondyle. Fixation should occur at 30° flexion and in neutral rotation. A cortical fixation system would appear to be more suitable, to avoid the risk of tunnel convergence. When appropriately executed, lateral tenodesis not only reduces stress on the anterior cruciate ligament graft but also restores physiological knee kinematics.

## Figures and Tables

**Table 1:** Analysis of articles on the four criteria (femoral fixation, position during fixation, fixation method, relationship to the lateral collateral ligament (LCL)).

Studies	Femoral Location		Knee Position		Fixation Method	LCL Relationship	Study Design	Sample Size
	Proximo Distal	Antero Posterior	FLEXION	ER				
Xu et al. [31]	Proximal to LE	Posterior to LE	30°	0°	Screw or anchor	Superficial & Deep	Biomechanical	8
Puzzitiello et al. [21]	Proximal to LE	Anterior to LE	30°	0°	Screws	Deep	Technical note	NS
Jesani et al. [20]	Proximal to the femoral insertion of the lateral gastrocnemius	Anterior to femoral insertion lateral gastrocnemius	60°	0°	Staple + suture ^a^	Deep	Technical note	NS
Abusleme et al. [22]	Through septum		30°	0°	Suture ^a^	Deep	Technical note	NS
Temperato et al. [23]	Proximal to LE	Posterior to LE	30°	0°	Knotless anchor	Deep	Technical note	NS
Porter et al. [7]	Proximal to LE	NS	35°	NS	Tibial screw + suture ^a^	Deep	Cohort (II)	38
Da Silva Araújo et al. [24]	5 mm proximal to LE	5 mm posterior to LE	30°	0°	Suture ^b^	Superficial	Technical note	NS
Gomes et al. [25]	Proximal to LE	Posterior to LE	50–60°	RE	Screw + suture ^c^	Deep	Technical note	NS
Firoozabadi et al. [32]	31 mm proximal to LE	NS	90°	0°	Endobutton	Superficial	Technical note	NS
Anderson et al. [1]	Proximal to LE	Anterior to LE	NS	NS	Clip	Deep	Technical note	NS
Stuyts et al. [33]	Proximal to the femoral insertion of the popliteal tendon	Posterior to the femoral insertion of the popliteus tendon	60–90°	10–15°	Screws	Superficial	Technical note	NS
Bernholt et al. [34]	31.4 mm proximal to LE	NS	20°	0°	Clip	Deep	Technical note	NS
Goes et al. [35]	3 mm proximal to LE	3 mm posterior to LE	30°	0°	Screws	Deep	Technical note	NS
Wasdev et al. [36]	Distal Kaplan fibers: -1.5 cm proximal vessels -1.5 cm proximal fat pad -8 mm proximal LCL	Distal Kaplan fibers: -1.5 cm anterior LE vessels -1 cm posterior to fat pad -4 mm posterior to LCL	30°	0°	Screws	Deep	Technical note	NS
Bechis et al. [37]	ACL femoral tunnel exit		30°	0°	Suture ^b^	Deep	Technical note	NS
Schlichte et al. [38]	Proximal to LE (under physis)	Posterior to LE	30°	0°	Anchor + suture ^a^	Deep	Technical note	NS
Pavão et al. [39]	3 mm proximal to LE	3 mm posterior to LE	30°	0°	Screws	Deep	Technical note	NS
Behrendt et al. [40]	Proximal to LE	Posterior to LE	45–50°	0°	Screw or anchor	Deep or NS	Cohort (III)	52
Leyes-Vence et al. [41]	Proximal to LE	Posterior to LE	30°	RE	Suture ^b^	Deep	Technical note	NS
Moro et al. [42]	1 cm proximal to LE	1 cm posterior to LE	0°	0°	Screws	Deep	Technical note	NS
Koukoulias et al. [43]	Proximal to LE	Posterior to LE	10°	0°	Suture ^a,b^	Deep	Technical note	NS
Gali et al. [44]	31.4 mm proximal to epicondyle	NS	70°	0°	Screws	Superficial	Technical note	NS

FLE = flexion; ER = external rotation; LE = lateral epicondyle; ACL = anterior cruciate ligament; LCL = lateral collateral ligament; NS = not specified; a suture on itself; b suture with ACL transplant suture/endobutton; c suture to LCL.

## Data Availability

No new data were created or analyzed in this study.

## Supplementary Materials

The following are available online at https://www.mdpi.com/article/10.3390/jcm14217613/s1, Table S1: PRISMA 2020 Main Checklist. Ref. [62] is listed in the supplementary.

## Abbreviations

The following abbreviations are used in this manuscript:

ACL	Anterior cruciate ligament
ALL	Anterolateral ligament
LCL	Lateral collateral ligament
